# Regulation of Immunoproteasome Function in the Lung

**DOI:** 10.1038/srep10230

**Published:** 2015-05-19

**Authors:** Ilona E. Keller, Oliver Vosyka, Shinji Takenaka, Alexander Kloß, Burkhardt Dahlmann, Lianne I. Willems, Martijn Verdoes, Hermen S. Overkleeft, Elisabeth Marcos, Serge Adnot, Stefanie M. Hauck, Clemens Ruppert, Andreas Günther, Susanne Herold, Shinji Ohno, Heiko Adler, Oliver Eickelberg, Silke Meiners

**Affiliations:** 1 Comprehensive Pneumology Center (CPC), University Hospital, Ludwig-Maximilians University, Helmholtz Zentrum München, Member of the German Center for Lung Research (DZL), Munich, Germany; 2 Research Unit Protein Science, Helmholtz Zentrum München, Munich, Germany; 3 Institute of Biochemistry, Charité-Universitätsmedizin Berlin, Berlin, Germany; 4 Department of Bio-organic Synthesis, Leiden University, Leiden, The Netherlands; 5 INSERM U955, Département de Physiologie, Université Paris-Est Créteil (UPEC), Créteil, France; 6 Department of Internal Medicine, Justus-Liebig-University Giessen, Universities Giessen & Marburg Lung Center (UGMLC), Member of the German Center for Lung Research (DZL), Giessen, Germany; 7 Agaplesion Pneumologische Klinik Waldhof-Elgershausen, Greifenstein, Germany; 8 Department of Internal Medicine II, Section of Infectious Diseases, Justus- Liebig-University, Universities Giessen & Marburg Lung Center (UGMLC), Member of the German Center for Lung Research (DZL), Giessen, Germany; 9 Research Unit Gene Vectors, Helmholtz Zentrum München, Munich, Germany

## Abstract

Impaired immune function contributes to the development of chronic obstructive pulmonary disease (COPD). Disease progression is further exacerbated by pathogen infections due to impaired immune responses. Elimination of infected cells is achieved by cytotoxic CD8^+^  T cells that are activated by MHC I-mediated presentation of pathogen-derived antigenic peptides. The immunoproteasome, a specialized form of the proteasome, improves generation of antigenic peptides for MHC I presentation thereby facilitating anti-viral immune responses. However, immunoproteasome function in the lung has not been investigated in detail yet. In this study, we comprehensively characterized the function of immunoproteasomes in the human and murine lung. Parenchymal cells of the lung express low constitutive levels of immunoproteasomes, while they are highly and specifically expressed in alveolar macrophages. Immunoproteasome expression is not altered in whole lung tissue of COPD patients. Novel activity-based probes and native gel analysis revealed that immunoproteasome activities are specifically and rapidly induced by IFNγ treatment in respiratory cells *in vitro* and by virus infection of the lung in mice. Our results suggest that the lung is potentially capable of mounting an immunoproteasome-mediated efficient adaptive immune response to intracellular infections.

The lung is constantly exposed to acute environmental agents such as noxious gases, aerosols, and pathogens^1^. Efficient clearance and defense mechanisms are thus indispensable to protect the lung from injury and maintain lung function. Failure of these defense mechanisms results in sustained inflammation and activation of the immune system, contributing to chronic pulmonary diseases with impaired lung structure and function^2^. This is particularly evident for chronic obstructive pulmonary disease (COPD): lungs of COPD patients show increased levels of inflammatory cytokines such as tumor necrosis factor α (TNFα) and interferon-γ (IFNγ) as well as increased numbers of both innate and adaptive immune cells^2^,^3^. In addition, bacterial or viral infections in COPD patients often result in acute exacerbations and accelerate disease progression, suggesting that, amongst others, the adaptive immune system is unable to efficiently detect and eliminate infected lung cells to terminate pathogen amplification. Intracellular antigens are detected by pathogen-specific activated CD8^+^  T cells that patrol the lungs for pathogen-derived peptides presented in complex with major histocompatibility complex (MHC) I on the cell surface of infected cells.

The ubiquitin-proteasome system is the major peptide provider for MHC I antigen presentation. It degrades more than 90 % of all cellular proteins - including old and damaged ones - into small peptides^4^,^5^,^6^. The proteasome consists of a barrel-shaped 20S proteolytic core particle which is activated by different proteasome regulators to form for instance the 26S, which degrades poly-ubiquitinated proteins in an ATP-dependent manner, and hybrid proteasomes^7^. The 20S core is composed of four heptameric rings comprising α- and β-subunits with α_7_β_7_β_7_α_7_ structure. In standard proteasomes, three of the seven β-subunits - namely β1, β2, and β5 - exhibit proteolytic activity. A replacement of these β-subunits by their immunosubunit counterparts, i.e. low molecular mass protein (LMP) 2, multicatalytic endopeptidase complex-like 1 (MECL-1), and LMP7, also termed β1_i_, β2_i_, and β5_i_, respectively, results in formation of so-called immunoproteasomes. Immunoproteasomes are constitutively present in lymphoid cells but their synthesis can be induced rapidly also in non-immune cells by IFNγ, or TNFα, e.g. upon viral or bacterial infection^8^. The newly assembled immunoproteasomes have altered cleavage kinetics compared to their 20S standard counterparts and generate antigenic peptides that are preferentially presented by MHC I molecules^9^. As such, rapid and specific induction of immunoproteasomes is required for efficient elimination of infected cells via the adaptive immune system. Increasing evidence suggests impairment of proteasome function by smoke exposure and in COPD^10^,^11^,^12^,^13^, however, until now it is not known whether immunoproteasome function is affected as well. Moreover, cell-specific expression of immunoproteasomes in the lung has not been analyzed so far and it is unclear to which degree immunoproteasome activity can be induced upon virus infection *in vivo*.

In this study, we comprehensively characterized immunoproteasome function, i.e. activity, in the lung by dissecting IFNγ-mediated regulation of specific catalytic activities of the immunoproteasome in different respiratory cell types *in vitro* and upon MHV-68 infection of the lung *in vivo*.

## Results

### Immunoproteasome expression in the murine lung

**Figure 1:**
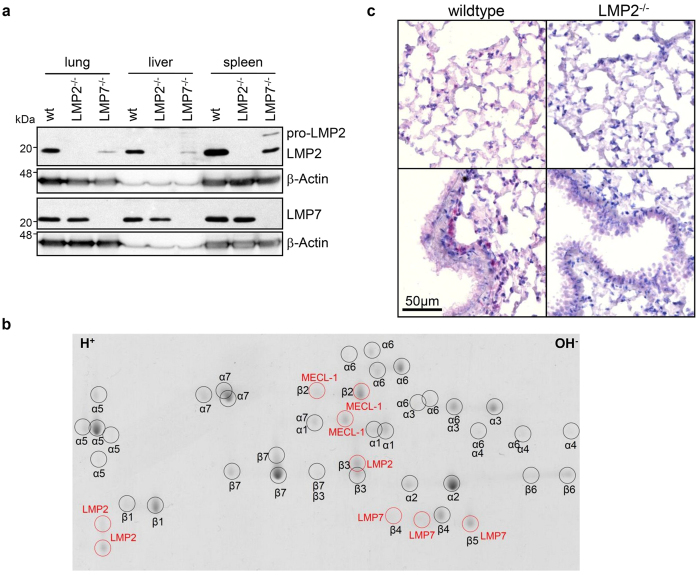
Immunoproteasome expression in mouse lungs. (**a**) Immunoproteasome expression in homogenates of whole lung, liver, and spleen in C57BL/6 wildtype (wt), LMP2^−/−^ or LMP7^−/−^ mice. (**b**) Coomassie stained 2D-gel of purified 20S proteasomes from C57BL/6 mouse lungs. Protein spots were identified by mass spectrometry, immunoproteasome subunits are indicated in red. (**c**) Immunohistochemistry analysis of LMP2 expression in wildtype and LMP2^−/−^ mice. Scale bar represents 50 μm.

As immunoproteasome expression in the lung has not been investigated in detail so far, we first examined total expression levels of immunoproteasome subunits in the murine lung compared to liver and spleen including tissues from LMP2 and LMP7 deficient mice as controls (Fig. 1a). Wildtype lungs contained intermediate amounts of the immunoproteasomal subunits LMP2 and LMP7 compared to liver and spleen. While LMP7 levels were unchanged in LMP2 deficient mice, LMP2 protein levels were evidently decreased in LMP7 deficient mice and the unprocessed pro-form of LMP2 accumulated in spleens of LMP7 deficient mice. To confirm incorporation of immunoproteasome subunits into active 20S proteasomes and their relative distribution compared to standard β-subunits, we next isolated active 20S proteasomes from the lungs of healthy wildtype mice via sucrose-gradient fractionation and separated them on a 2D gel (Fig. 1b and Supplementary Fig. S1). By mass spectrometry of excised spots, we detected all different 14 subunits of the 20S proteasome. All three immunoproteasome subunits were present in addition to the three standard proteasome subunits β1, β2, and β5. Each immunoproteasome subunit was identified from three distinct spots, indicating post-translational modifications of these subunits.

To specify the pulmonary cell types that contain immunoproteasomes, we stained murine lungs with LMP2- and LMP7-detecting antibodies using lungs of the respective knockout animals as controls. Although we tested several commercially available antibodies, immunohistochemical detection of LMP7 proved to be unspecific as controlled by lungs of LMP7 knockout mice while staining for LMP2 was specific (Fig. 1c): Alveolar epithelial and parenchymal cells of the lung expressed only very low amounts of LMP2, whereas alveolar macrophages were highly positive for this immunoproteasome subunit. Individual cells in the vicinity of airways also exhibited prominent LMP2 staining.

### Immunoproteasome expression is not altered between donor and COPD lungs

**Figure 2:**
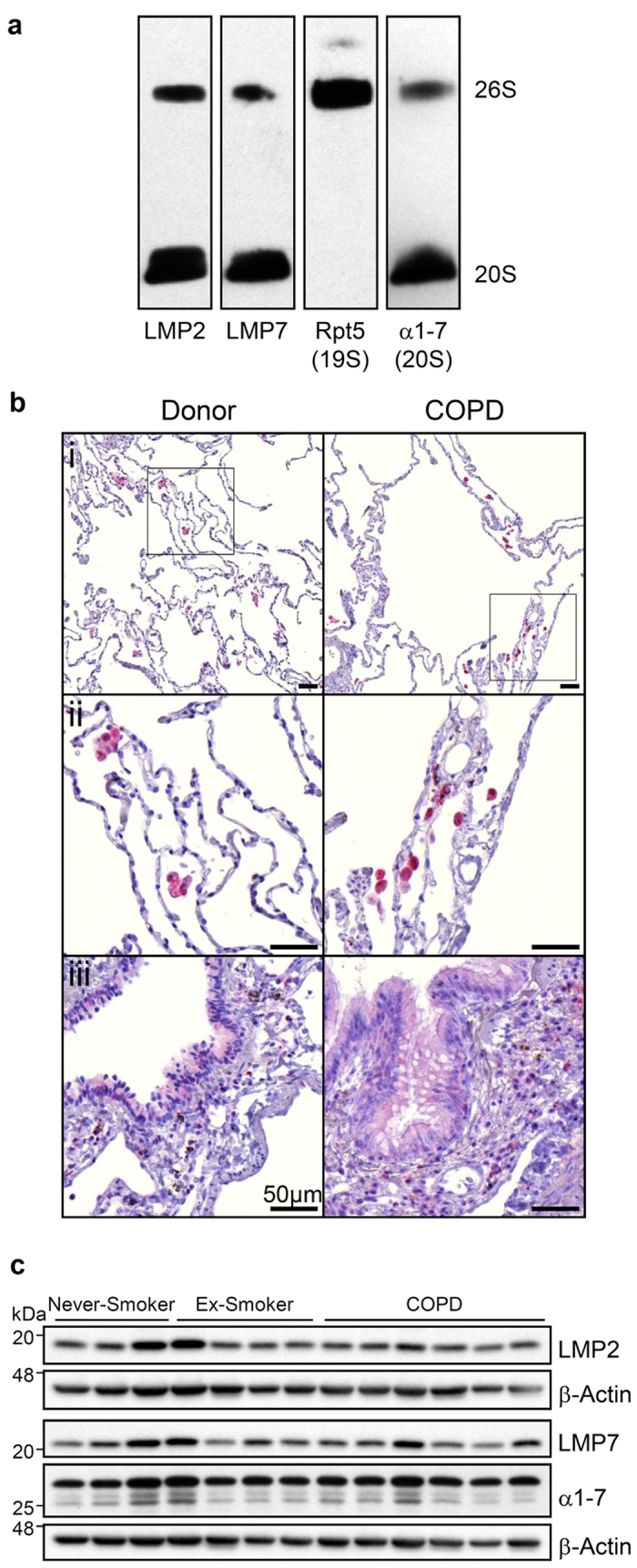
Immunoproteasome expression in human donor and COPD lungs. (**a**) Immunoproteasome expression in human donor lung lysate under native conditions. Native gels were blotted and LMP2, LMP7, α1-7 subunits (20S), Rpt5 (19S) was detected with respective antibodies. (**b**) LMP2 staining of human lung sections from donors (n = 5) and COPD (n = 9) patients: (i) alveolar parenchyma, (ii) alveolar macrophages, (iii) bronchial epithelium with goblet cell hyperplasia in COPD. Scale bar represents 50 μm. (**c**) Protein expression of immunoproteasome subunits LMP2 and LMP7 and total 20S (α1-7) proteasomes in lungs of human organ donors (never-smoker or ex-smoker) and COPD patients.

In a next step, we thoroughly investigated immunoproteasome expression in the human lung by using native PAGE and immunoblotting of human donor lung tissue. We unambiguously identified the immunoproteasome subunits LMP2 and LMP7 mainly in active 20S but also to some extent in 26S fractions as confirmed by blotting for respective proteasomal 19S (Rpt5) and 20S subunits (α1-7) (Fig. 2a).

Immunohistochemical analysis of LMP2 in end-stage COPD tissue (GOLD stage III and IV) from explanted lungs revealed no obvious alteration in cell-type specific expression of LMP2 compared to lungs from human donors (Fig. 2b). We observed prominent but variable staining for LMP2 mainly in alveolar macrophages. While bronchial epithelial cells showed some positive staining, alveolar epithelial cells were negative for LMP2. In addition to alveolar macrophages, cells in the vicinity of airways also showed some LMP2 reactivity. However, our immunohistochemical staining was heterogeneous and did not allow a reliable quantification of LMP2 expression levels in lung tissue samples of COPD patients compared to controls.

In human lung tissue homogenates from cancer resections of never-smokers, ex-smokers, and COPD GOLD stage I and II classified patients, we again did not observe any significant difference in the levels of LMP2 and LMP7 between these groups (Fig. 2c). Of note, expression of the 20S proteasome was also not altered in COPD tissue compared to non-COPD controls, indicating that the proteasome is not obviously dysregulated in these samples.

### Active immunoproteasomes are induced by IFNγ in parenchymal cells of the lung

**Figure 3:**
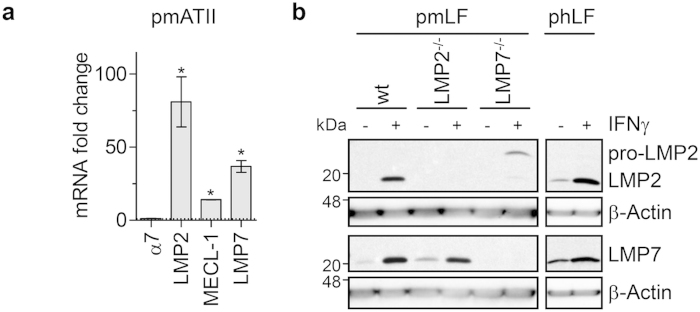
Immunoproteasomes are induced by IFNγ in lung parenchymal cells. (**a**) mRNA fold change of 20S α7-subunit and immunoproteasome subunits in primary mouse alveolar type II cells (pmATII) after 24 h of IFNγ treatment compared to control. Results are combined data from three independent experiments (mean +/− SEM, Mann-Whitney-U test, * = *p* < 0.05). (**b**) Western Blot showing induction of LMP2 or LMP7 in primary mouse (wildtype (wt), LMP2^−/−^, or LMP7^−/−^; pmLF) and human donor lung fibroblasts (phLF) after 24 h of IFNγ treatment. Results are representative for two independent experiments.

With the basal expression levels of immunoproteasomes being low in parenchymal cells, but high in immune cells of the lung, we next investigated to what extent immunoproteasomes can be induced in parenchymal cells by IFNγ, which has been shown as a major cytokine involved in acute virus infection and a major inducer of immunoproteasomes^14^. We confirmed IFNγ-mediated induction of immunoproteasomes in primary parenchymal cells of the murine and human lung: IFNγ strongly induced immunoproteasomal gene expression in mouse primary alveolar type II cells (pmATII) after 24 h of treatment (Fig. 3a). LMP2 and LMP7 protein levels were both strongly induced in primary human (phLF) and mouse lung fibroblasts (pmLF) after IFNγ stimulation for 24 h (Fig. 3b). Similar to tissue homogenates shown in Fig. 1a, LMP7 was induced in fibroblasts from LMP2 deficient mice to the same degree as in wildtype mice, but the unprocessed pro-LMP2 accumulated in LMP7 deficient fibroblasts.

**Figure 4:**
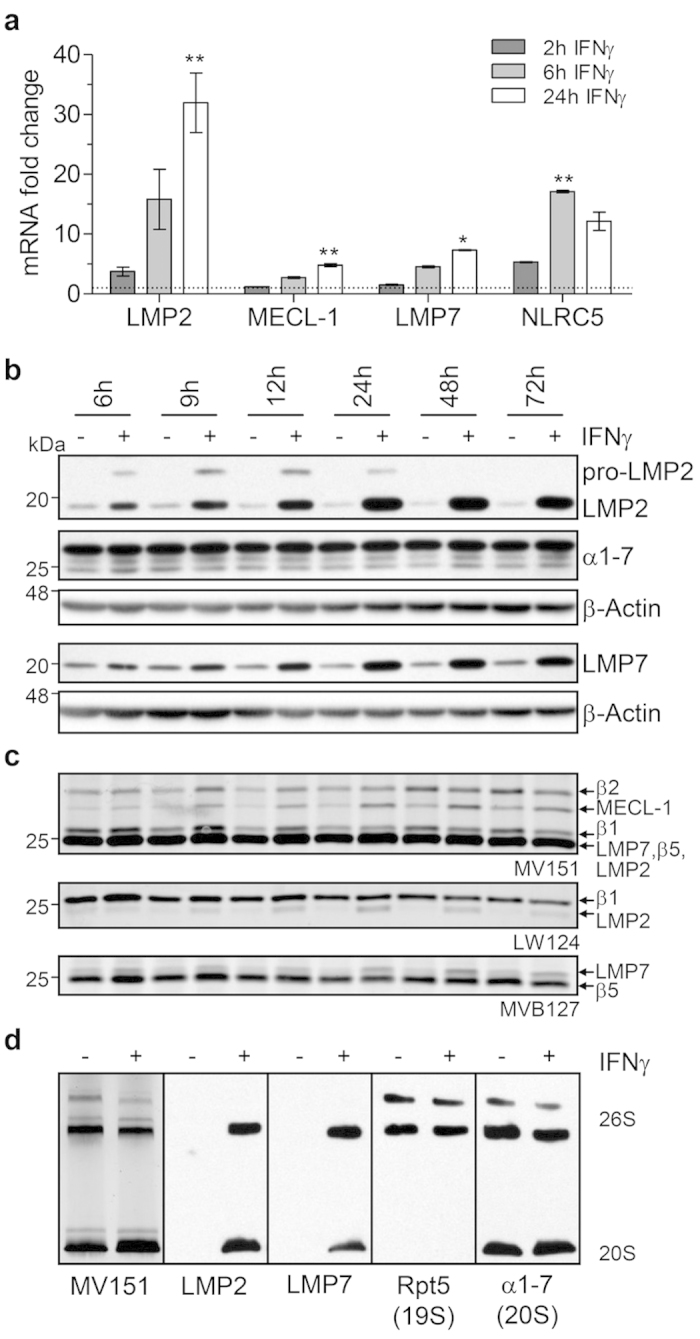
Immunoproteasome induction kinetics in alveolar epithelial cell line. (**a**) mRNA fold change of immunoproteasome subunits and their transcriptional activator NLRC5 in response to IFNγ (75 U/ml) after 2, 6, or 24 h in A549 cells. Results are the combined data of three independent experiments (mean +/− SEM, Kruskal-Wallis Test with Dunn’s Post Test, * = *p* < 0.05, ** = *p* < 0.01). (**b**) Time course of expression of immunoproteasome subunits LMP2 and LMP7 and total 20S α-subunits in native lysates of A549 cells from 6 up to 72 h after IFNγ treatment. Results are representative for three independent experiments. (**c**) Fluorescent ABP labeling of the same lysates as in (**b**) with MV151 (labeling all active β-subunits), LW124 (β1 and LMP2 specific) or MVB127 (β5 and LMP7 specific). Results are representative for three independent experiments. (**d**) Native gel analysis of A549 lysates +/− IFNγ treatment for 72 h: MV151-ABP analysis and Western Blot of native lysates with LMP2 and LMP7 antibodies. α1-7 was used to detect 20S complexes, Rpt 5 (19S subunit) was used to detect 26S proteasome complexes. Results are representative for three independent experiments.

To define the kinetics and activities of newly formed immunoproteasomes after IFNγ stimulation in detail, we treated the human alveolar epithelial cell line A549 from 2 up to 72 h with IFNγ. mRNA levels of all three immunoproteasome subunits were upregulated after 2 h and further increased up to 24 h in A549 cells upon IFNγ treatment (Fig. 4a). Transcript levels of NLRC5, a recently identified transactivator of LMP2 and MHC class I genes^15^, transiently peaked at 6 h but declined after 24 h (Fig. 4a).

Protein levels of both LMP2 and LMP7, were upregulated already after 6 h and stayed elevated until 72 h after IFNγ treatment (Fig. 4b). The unprocessed pro-form of LMP2, which indicates that the protein is not yet incorporated into mature 20S proteasome complexes^16^, was detected between 6 and 24 h of IFNγ treatment suggesting that LMP2-containing immunoproteasomes are only finally assembled 48 h after IFNγ stimulation. As total 20S proteasome levels, however, were not altered, these results indicate a shift from standard 20S towards immunoproteasome expression in IFNγ-exposed lung alveolar cells (Fig. 4b). *De novo* assembly of active immunoproteasomes was further proven by use of activity-based probes (ABP). ABPs covalently bind to and label only active catalytic β-subunits of the intact 20S catalytic core^17^. Here, we made use of three distinct site-specific ABPs that allowed us to discriminate the active standard and immunoproteasome subunits^18^. Native lysates of IFNγ-treated A549 were incubated with the respective ABPs and then separated under denaturing conditions to quantify the labeled catalytic subunits of the proteasome: The activity of all three immunoproteasome subunits, LMP2, MECL-1, and LMP7, increased up to 24 h and slightly decreased at 72 h after IFNγ treatment while the standard catalytic subunits β1, β2, and β5 were inversely regulated after an initial 24 h activation burst (Fig. 4c). The novel technique of ABP detection in native gels revealed ABP labeling of five different active 26S and hybrid proteasome complexes with a slight shift from active 26S to 20S proteasomes after 72 h of IFNγ treatment (Fig. 4d). LMP2 and LMP7 were incorporated into both, active 20S and 26S complexes, as shown by immunoblotting.

### Active immunoproteasomes are induced by MHV-68 infection in the lung

**Figure 5:**
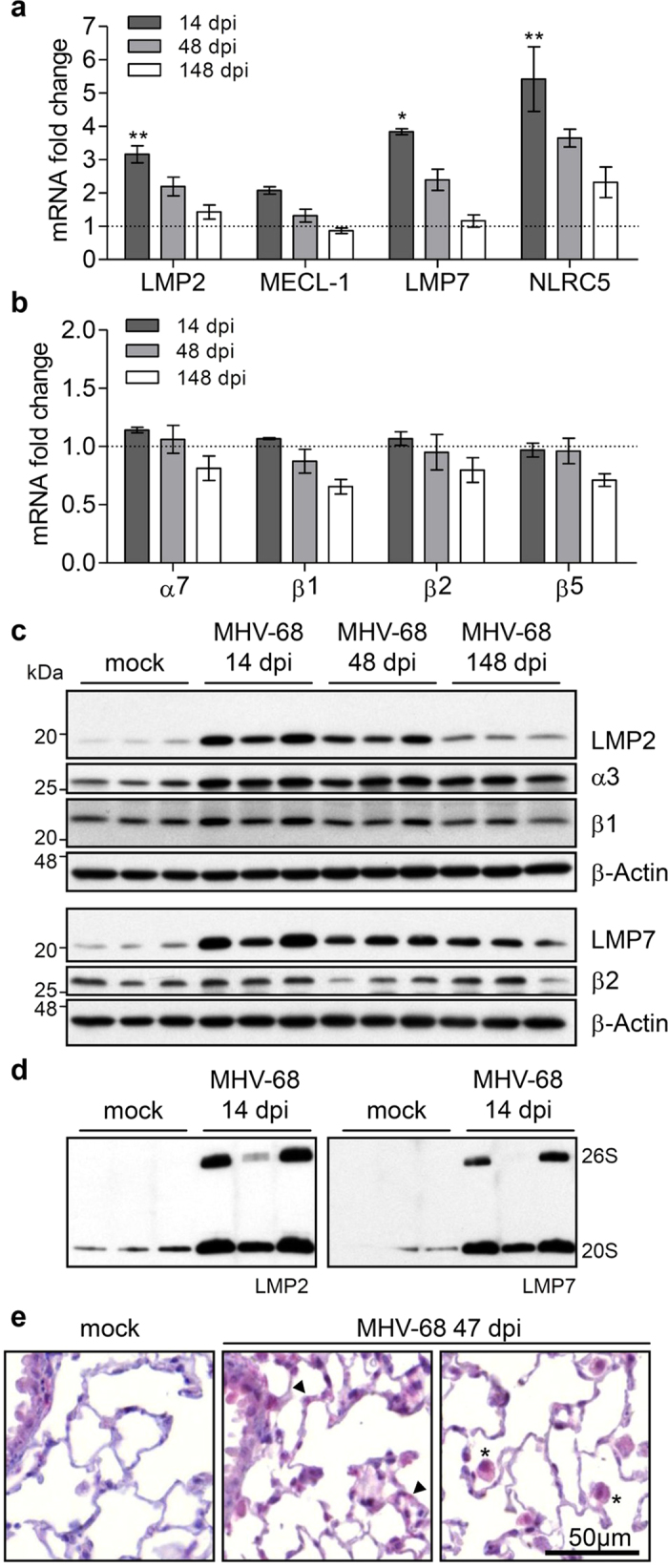
Murine gammaherpesvirus-68 (MHV-68) infection induces immunoproteasomes in the lung. (**a**) and (**b**) relative mRNA levels of standard proteasome subunits α7, β1, β2, β5 and immunoproteasome subunits LMP2, MECL-1, LMP7 and MHC I gene transactivator NLRC5 in the lungs of MHV-68 infected mice (day 14, 48 and 148 post infection) compared to mock-infected controls, Rpl19 served as housekeeping gene, n = 3 per group (mean +/− SEM, Kruskal-Wallis Test with Dunn’s Post Test, * = *p* < 0.05, ** = *p* < 0.01). (**c**) Western Blot analysis of LMP2, LMP7, α3, β1 and β2 protein expression of whole lung homogenate of MHV-68 infected mice (day 14, 48 and 148) compared to uninfected controls. (**d**) Native Western Blot of lung lysates from uninfected or MHV-68 infected mice after 14 days. (**e**) Immunohistochemistry analysis of LMP2 expression in wildtype lung slices at 47 dpi. All results are representative for two independent experiments. dpi, days post infection.

As IFNγ-mediated induction of immunoproteasome is indispensable for efficient antigen presentation of viral proteins during infection, we investigated the kinetics of immunoproteasome expression and activity in the lung after murine gammaherpesvirus-68 (MHV-68) infection *in vivo*. MHV-68 infection strongly induced immunoproteasome expression: mRNA levels of all three immunoproteasome subunits were highest at day 14 post infection and declined to control levels after 148 days, even though IFNγ and TNFα transcript levels were still increased at that time point (Fig. 5a and Supplementary Fig. S3a). NLRC5 mRNA levels showed similar expression kinetics, but were still elevated after 148 days. Expression of standard proteasome subunits was not obviously altered upon infection and even slightly decreased over time (Fig. 5b).

On the protein level, immunoproteasomes were strongly induced after 14 days and were still found to be slightly elevated 148 days after infection (Fig. 5c). The α3 as well as the β1 and β2 constitutive subunits were also increased after 14 days of infection, although to a lesser extent (Fig. 5c). The inducible immunoproteasome subunits LMP2 and LMP7 were found in both, 20S and 26S, complexes, as determined by native gel immunoblotting (Fig. 5d). LMP2 staining of virus-infected mouse lungs revealed that the overall increase of LMP2 protein levels after 47 days was mainly attributable to enhanced LMP2 expression in alveolar epithelial cells and alveolar macrophages (Fig. 5e).

**Figure 6:**
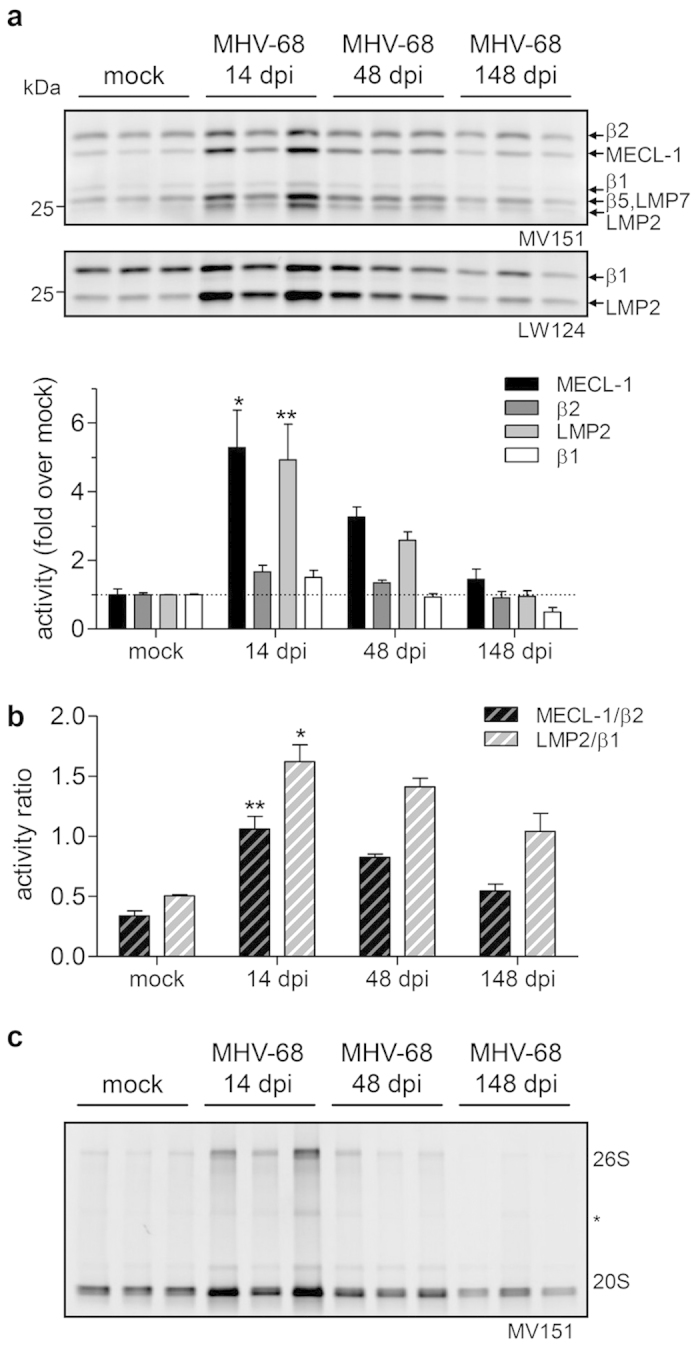
Immunoproteasome and standard proteasome activities in the lung during course of MHV-68 infection. (**a**) Activity-based probe labeling of native whole lung lysates of infected mice (mock, 14, 48, 148 dpi) with MV151 (labeling all active β-subunits), LW124 (β1 and LMP2 specific) and densitometric analysis of MECL-1, β2, LMP2 and β1, depicted as fold increase over uninfected mice. (**b**) Activity ratios of intensities of MECL-1/β2 and LMP2/β1. (**c**) Native gel analysis of lung lysates labeled with activity-based probe MV151. (mean +/− SEM, Kruskal-Wallis Test with Dunn’s Post Test, * = *p* < 0.05, ** = *p* < 0.01). All results are representative for two independent experiments. dpi, days post infection.

ABP labeling of native lung lysates of infected mice revealed that the specific activity of LMP2 and MECL-1 was transiently increased during the course of infection and normalized to control levels after 148 days (Fig. 6a). In these mouse samples, we were not able to discriminate LMP7 and β5 activities as both mouse subunits have a similar molecular weight (Fig S2), different from the human subunits (Fig. 4). Of note, activity of standard subunits β1 and β2 was also increased, but to a lesser extent than their respective immunoproteasome subunit counterparts LMP2 and MECL-1 (Fig. 6a). The pronounced rise in specific immunoproteasome activity during the course of virus infection closely followed a transient increase in total proteasome activity with similar kinetics and resulted in a considerable shift from standard to immunoproteasome activity in these samples (Fig. 6b and Supplemental Fig. S3b).

The increase in total proteasome activity was attributable to both 20S and 26S complexes by analysis of native PAGE of ABP-labeled lysates (Fig. 6c). Taken together, our data show prominent induction of active immunoproteasomes in the lung by IFNγ in different alveolar cell types and by virus infection *in vivo* indicating that these cells are able to mount efficient immunoproteasome-mediated immune responses to infection. Of note, while the kinetics of specific immunoproteasome activities were similar to the transcript kinetics of immunoproteasome subunits, activation of standard proteasome activity upon acute virus infection did not involve transcriptional regulation but appears to take place on the post-transcriptional level.

## Discussion

Immunoproteasomes play a pivotal role in MHC I antigen presentation. We thus investigated the function and plasticity of immunoproteasomes in human and mouse lungs as well as upon virus-infection.

Protein expression levels of immunoproteasomes in the mouse lung were comparable to those in the liver but lower than in mouse spleen. While we could specifically detect LMP2 and LMP7, immunodetection of the third immunoproteasome subunit MECL-1 was unspecific with several commercially available antibodies. Biochemical purification of lung 20S proteasomes revealed incorporation of both standard and immunoproteasome subunits into active 20S complexes. Of note, each of the three immunoproteasome subunits was found in three distinct protein spots indicating post-translational modifications or isoform expression in the mouse lung. However, our mass spectrometry analysis did not allow us to identify any modifications, which was beyond the scope of this project. Further analysis of native proteasome complexes in the lung by blotting of native PAGE gels revealed that 20S immunoproteasomes can be found both in the 20S and 26S proteasome fraction of mouse and human lungs (Figs. 2a, 4d and 5d), implying that immunoproteasomes contribute to both ubiquitin-dependent (26S) and -independent (20S) degradation of proteins.

While other studies have examined total levels of immunoproteasomes in human tissue, whole rat lungs, and in LCMV-infected mouse lungs and other organs^19^,^20^,^21^,^22^,^23^, cell-specific expression in the lung has not yet been investigated^23^,^24^. Here, we show that the immunoproteasomal subunit LMP2 is expressed at low basal levels in lung parenchymal cells (alveolar type I and II cells, fibroblasts) and the bronchial epithelium but strongly expressed in alveolar macrophages (Figs. 1c and 2b). Specificity of our staining was confirmed in control lungs of LMP2 deficient mice (Fig. 1c). This is in line with a recent study that demonstrated expression of LMP2 and LMP7 in lung granulomas of sarcoidosis patients^24^. Our *in vitro* data show that primary alveolar type II cells and fibroblasts have the capability to express immunoproteasomes after IFNγ stimulation, thus enabling immunoproteasome-dependent antigen presentation. Interestingly, fibroblasts from LMP7 deficient mice express some unprocessed LMP2 compared to wildtype mice. It was previously shown that LMP7 is necessary for efficient incorporation of LMP2^25^, which explains our observation of the presence of unprocessed LMP2 both after stimulation with IFNγ *in vitro* (Fig. 3b) as well as in spleen homogenates of LMP7 deficient mice (Fig. 1a). In A549 cells, the pro-form of LMP2 was detected until 24 h after IFNγ treatment, while pro-LMP7 was not detectable at any time-point (even though the LMP7 antibody we used detects both the unprocessed and mature form of LMP7). This might be due to preferential incorporation of pro-LMP7 into 20S compared to its standard proteasome counterpart pro-β5, as suggested previously^26^,^27^.

Using a novel and specific set of activity-based probes^18^, we were able to dissect the six different active sites of the standard and immunoproteasome 20S, which specified immunoproteasome function in the lung. This is not possible with commercially available proteasome substrates. A striking feature of these activity-based probes is that beyond quantification of the three main proteasomal activities, we can specifically discriminate activities for the standard and the respective immunoproteasome subunit counterparts β1/LMP2, β2/MECL-1, and β5/LMP7 as they are labeled within the same lysate (Fig. 4c). The novel combination of activity-based probe labeling of all catalytic active sites of the proteasome with native gel electrophoresis permitted us to assign newly assembled immunoproteasomes to active 20S and 26S proteasome complexes (Fig. 4d). Using this innovative biochemical toolbox, we showed that IFNγ can rapidly induce expression and assembly of active immunoproteasomes in parenchymal cells of the lung. This cannot be achieved with conventional and commercially available proteasome activity assays. With these techniques at hand, we also assessed immunoproteasome activity in the course of virus infection of the lung. For that, we used the model of MHV-68, since intranasal infection of mice leads to productive virus replication in the lung accompanied by virus-induced cell damage and subsequent development of pulmonary fibrosis^28^,^29^. Viral infections induce immunoproteasomes via IFNγ as part of the adaptive immune response to infections^30^ thereby facilitating the specific detection and targeted elimination of infected cells by the immune system: Pathogenic, e.g. viral, proteins are cleaved by immunoproteasomes into antigenic peptides for MHC I presentation^14^,^31^,^32^,^33^,^34^. MHC I epitopes are then recognized by specific cytotoxic CD8^+^ T cell clones that kill infected cells. To raise a specific clonal T cell response, antigen presenting cells (APC) in the lung take up pathogens and migrate to the lymph nodes to prime CD8^+^ T cells. Importantly, APCs and infected parenchymal cells need to present the same MHC I epitope to prime an efficient clonal CD8^+^ T cell response, respectively. As APCs constitutively express immunoproteasomes, IFNγ-mediated upregulation of immunoproteasomes in infected parenchymal cells is thus indispensable for mounting an efficient immune response against the pathogen^14^,^31^,^32^,^33^,^34^,^35^. In our MHV-68 infection model (Figs. 5 and 6), we were able to detect increased immunoproteasome transcript and protein levels which were highest 14 days after viral infection. While mRNA transcripts were back at baseline at day 148, protein levels of LMP2 and LMP7 were still increased, suggesting proteasome stabilization and extended half-life of proteasomes after infection. This might also explain the observation of slightly increased standard proteasomes on the protein and activity levels at day 14, which cannot be explained by increased transcript levels (Figs. 5 and 6). Total proteasome activity, as assessed by fluorescent activity-based probes, was transiently increased up to twofold during infection and was back at baseline after 148 days. The increase in proteasome activity was attributed to an increase in both 20S and 26S activities which both comprised the virus-induced immunoproteasome subunits LMP2 and LMP7. These data suggest ubiquitin-dependent and -independent degradation of proteins by immunoproteasomes during infection.

Over the course of infection, we observed a shift in standard versus immunoproteasome activity in MHV-68 infection, which was resolved for the catalytic subunit pair MECL-1/β2, but not for LMP2/β1 subunits (Fig. 6b). This indicates that virus infection has a long-term effect on antigen processing by immunoproteasomes. In part, this might be explained by the nature of MHV-68 infection, which can persist latently in lung epithelial cells and macrophages^36^,^37^ and can be spontaneously reactivated. An indicator for such reactivation is the still increased level of IFNγ 148 days post infection.

COPD is characterized by loss of parenchymal tissue, chronic bronchitis, and bacterial colonization of the lower airways^2^. Respiratory infections exacerbate COPD pathology. Smokers and COPD patients suffer longer from respiratory infections and need more time to resolve them^38^. Accordingly, it has been shown that cigarette smoke, the main risk factor for COPD, generally dampens the host’s immune system in response to infections as it interferes with STAT-1 and IRF-3 immune signaling^39^,^40^,^41^,^42^,^43^. Cigarette smoke has also been shown to affect adaptive immune responses such as MHC II antigen presentation^2^,^44^. The role of the MHC I antigen presentation machinery in COPD in general and in viral exacerbations in particular has not been investigated so far. In this study, we did not detect increased levels of immunoproteasomes in early-staged COPD lungs (Fig. 2c). While Fujino *et al.* observed increased LMP2 and LMP7 transcript levels in primary alveolar type II cells of patients with early COPD stages, a recent study observed no differential expression of immunoproteasomes in lungs of end-stage COPD patients compared to controls^23^,^45^. This accords with our immunohistochemical analysis of end-stage-diseased COPD tissue, which did not reveal upregulation of the immunoproteasomal LMP2 subunit in alveolar epithelial cells. High immunoproteasome expression in alveolar macrophages, as observed here, may also account for extracellular immunoproteasomes in the BAL fluid of patients with acute respiratory distress syndrome^46^. We also did not observe any consistent change in standard versus immunoproteasome activities in early and late stage COPD lungs (data not shown). Overall proteasome activity has been assessed previously in COPD lungs using conventional proteasome activity assays with conflicting results: While Baker *et al.* did not observe significantly altered levels and activities in COPD lungs, Malhotra *et al.* reported that proteasome expression and activity declined and strongly associated with the severity of lung dysfunction in COPD patients^12^,^23^. However, as the corresponding author has recently expressed his concern on anomalies in figures in this article, this study has to be considered with caution^47^. It is well established though that proteasome activity can be impaired by cigarette smoke which may then add to development and progression of COPD^10^,^11^.

In this study, we show that lung parenchymal cells express immunoproteasomal subunits at low basal levels, but they can be rapidly induced to form active immunoproteasomes upon IFNγ stimulation *in vitro* or MHV-68 infection *in vivo*. This suggests that the lung is potentially capable of mounting an efficient adaptive immune response to intracellular infections.

## Methods

### Human lung tissue

For protein extraction, human lung tissue from never-smokers (n = 3), ex-smokers (n = 4), and COPD patients (n = 6) undergoing lung resection surgery for localized lung tumors was collected as previously described^48^. This study was approved by the institutional review board of the Henri Mondor Teaching Hospital (Créteil, France; AFSSAPS reference number B90895-60). All patients and control subjects signed an informed consent document before study inclusion. Paraffin-embedded lung sections of human lung transplant donors (n = 5) or COPD patients (n = 9) with end-stage disease were obtained from the Department of Thoracic Surgery in Vienna, Austria, as described elsewhere^49^. The study protocol was approved by the Ethics Committee of the Justus-Liebig-University School of Medicine (No. 31/93, 84/93, 29/01) and the University of Vienna Hospital ethics committee (EK-Nr 076/2009).

### Animals

Tissues or cells were isolated from C57BL/6 wildtype (Charles River Laboratories), LMP2^−/−^ (Psmb9^tm1Stl,^ 50), or LMP7^−/−^(Psmb8^tm1Hjf,^ 51) mice. All animal procedures were conducted according to international guidelines and with approval of the Bavarian Animal Research Authority in Germany. All surgery was performed under ketamine/xylazine anesthesia, and all efforts were made to minimize suffering.

### Virus infection of mice

8-12 week old female C57BL/6 mice were anesthetized using ketamine/xylazine and infected intranasally with 5 × 10^4^ plaque forming units (PFU) of murine gammaherpesvirus-68 (MHV-68) as described elsewhere^52^. Animals were sacrificed after 14, 48, or 148 days, uninfected mice served as controls and were sacrificed together with the 14 days infected mice, the group size was three per group. Mice were housed in individually ventilated cages during the MHV-68 infection period. All animal experiments were in compliance with the German Animal Welfare Act, and the protocol was approved by the local Animal Care and Use Committee (District Government of Upper Bavaria; permit number 124/08).

### Cell culture and reagents

The human A549 alveolar epithelial cell line was obtained from ATCC (ATCC® CCL-185™, American Type Culture Collection, Manassas, VA, USA). Cells were cultured in DMEM (21885025, Life Technologies, Carlsbad, CA, USA) supplemented with 10 % fetal bovine serum (FBS, P30-3702, PAN Biotech, Aidenbach, Germany) and 100 U/ml of Pen/Strep (15070063, Life Technologies) and cells were grown at 37 °C in a humidified atmosphere containing 5 % CO_2_. Human or mouse recombinant IFNγ (11040596001/11276905001, Roche, Basel, Switzerland) was used at concentrations of 75 U/ml.

### Activity-based probe labeling

Activity of the constitutive and immunoproteasome subunits was monitored by using a set of activity-based probes (ABP)^53^. The pan-reactive proteasome ABP MV151^17^ was used for assessing of β2/MECL-1 activities, LW124 for β1/LMP2 activity, and MVB127 was used to label β5/LMP7^18^. Hypoosmotic native lysates of total lung or A549 cells were diluted to a total protein concentration of 0.5 μg/μl with reaction buffer (50 mM HEPES pH 7.4, 100 mM KCl, 10 mM MgCl_2_). By shaking at 37 °C for 1 h, 30 μl of sample was incubated with 0.5 μM MV151, 0.25 μM LW124 or 1 μM MVB127, respectively, and subsequently quenched by the addition of 6x Laemmli (50 % v/v glycerol, 300 mM Tris·HCl, 6 % w/v SDS, 325 mM DTT, 0.1 % w/v bromophenol blue, pH 6.8) or 5x native loading buffer (50 % v/v glycerol, 250 mM Tris, 0.1 % w/v bromophenol blue, pH 7.5) to a final 1x concentration. Samples were separated on 15 % Tris-glycine SDS polyacrylamide gels or non-denaturing 3–8 % Tris-Acetate gels (Life Technologies) and active proteasome subunits were visualized using a fluorescent scanner (Typhoon TRIO+; Amersham biosciences). Images were taken at 450 PTM and 50 μm pixel resolution with fluorescence Cy3/TAMRA for ABPs MV151 and MVB127 while the Cy2 florescent channel was used for LW124 and analyzed by using ImageJ software. Equal sample loading was verified by staining gels with PageBlue^TM^ (24620, Fisher Scientific, Schwerte, Germany).

### Statistics and Software

Data were analyzed with Image Lab^TM^ (Version 3.0.1., Bio-Rad, Hercules, CA, USA), ImageJ (ImageJ, U. S. National Institutes of Health, Bethesda, Maryland, USA), or Prism5 (Version 5.0, GraphPad Software, Inc., La Jolla, CA, USA). Statistics were performed using Prism5 with non-parametric tests and appropriate *post hoc*-analysis. *P*-values < 0.05 were considered statistically significant.

Additional detail on the methods is provided in an online data supplement.

## Author Contributions

I.E.K. and S.M. conception and design of research; L.I.W., M.V., H.S.O., E.M., S.A., C.R., A.G., S.O. and H.A. provided (clinical) samples and reagents; I.E.K., O.V., S.T., A.K. and S.O. performed experiments; I.E.K., O.V., S.T., A.K., B.D., S.M.H. and H.A. analyzed data; I.E.K., O.V., S.T., A.K., B.D., S.M.H., S.H., H.A. and S.M. interpreted results; I.E.K. prepared figures; I.E.K. and S.M. drafted manuscript; I.E.K., O.V., B.D., S.M.H., S.H., H.A., O.E. and S.M. edited and revised manuscript; all authors approved final version.

## Additional Information

**How to cite this article**: Keller, I. E. *et al.* Regulation of Immunoproteasome Function in the Lung. *Sci. Rep.* **5**, 10230; doi: 10.1038/srep10230 (2015).

## Supplementary Material

Supplementary Information
