# Supplementary material for: Regulation of Immunoproteasome Function in the Lung
Source: Sci Rep. 2015 May 19;5:10230. doi: 10.1038/srep10230 (PMC4437306; doi:10.1038/srep10230)
Supplement: Supplementary Information [file srep10230-s1.pdf]

Supplementary information for the manuscript

Regulation of Immunoproteasome Function in the Lung

Ilona E. Keller, Oliver Vosyka, Shinji Takenaka, Alexander Kloß, Burkhardt Dahlmann, Lianne I. Willems, Martijn Verdoes, Hermen S. Overkleeft, Elisabeth Marcos, Serge Adnot, Stefanie M. Hauck, Clemens Ruppert, Andreas Günther, Susanne Herold, Shinji Ohno, Heiko Adler, Oliver Eickelberg, Silke Meiners

SUPPLEMENT METHODS

Primary lung fibroblast isolation:

Primary mouse or human lung fibroblasts were isolated as described[1]. Mouse fibroblasts were used between passages 2-4, human fibroblasts before passage 6.

Mouse alveolar epithelial cell isolation and culture:

Primary alveolar type II cells (pmATII) were isolated from C57BL/6 mice as described previously[2].

Protein extracts and Western Blotting: Cells or dismembrated frozen tissue was lysed in ice-cold RIPA buffer (50 mM Tris·HCl, 150 mM NaCl, 1% Nonidet P-40, 0.5% sodium deoxycholate, and 0.1% SDS, pH 7.5), supplemented with protease inhibitor cocktail cOmplete (Roche, Basel, Switzerland). After 20 min incubation on ice, lysates were centrifuged at maximum speed for 20 min at 4°C and supernatants were used for further analysis.

To prepare native lysates, samples were resuspended in distilled water containing cOmplete protease inhibitors and subjected to five cycles of freezing (liquid N_2) and thawing (37°C waterbath). Cell debris was removed by centrifugation as described above. Protein concentrations were assessed using Pierce BCA kit (Thermo Fisher Scientific, Waltham, MA, USA). Western Blot analysis was performed as described[3]. Antibodies directed against LMP2 (1:1,500, ab3328, polyclonal), LMP7 (1:1,500, ab3329, polyclonal), PSMA4 (α3, 1:1,000, ab119419, clone 1H10) or 20S alpha 1+2+3+5+6+7 (α1-7, 1:1,000, ab22674, clone MCP231) were from Abcam (Cambridge, UK). An HRP-coupled antibody detecting β-Actin (1:40,000, A3854, clone AC-15) was purchased from Sigma-Aldrich (St. Louis, MO, USA), the antibody detecting the 19S subunit Tbp1 (Rpt5, 1:3,000, A303-538A, polyclonal) was purchased from Bethyl Laboratories (Montgomery, TX, USA). Antibodies detecting 20S

proteasome subunits β1 and β2 were from Santa Cruz (β1: 1:500, sc-67345, polyclonal; β2: 1:500, sc-58410, clone MCP165; Santa Cruz Biotechnology, Inc., Dallas, TX, USA)

Quantitative real-time RT-PCR: Total RNA from cells was isolated using Roti®-Quick-Kit (Carl Roth, Karlsruhe, Germany). 100-1,000 ng per sample of total RNA were reverse-transcribed using random hexamers (Life Technologies, Carlsbad, CA, USA) and M-MLV reverse transcriptase (Sigma-Aldrich). Quantitative PCR was performed using the SYBR Green LC480 System (Roche Diagnostics, Mannheim, Germany), gene-specific primer sequences are listed in Table S1.

Immunohistochemistry: Human or mouse lung sections (3 µm) were deparaffinized in Xylene and rehydrated. Slides were incubated in solution containing 80% methanol and 1.8% H_2O_2 for 20 min to quench endogenous peroxidase activity. Heat-induced antigen retrieval was performed in 0.05% citraconic anhydride buffer (pH 7.4). Slides were washed with TBST buffer (20 mM Tris, 135 mM NaCl, 0.02% Tween, pH 7.6), blocked with Rodent Block M (Biocare, Concord, CA, USA) for 30 min, washed and incubated for 60 min with an LMP2 specific antibody (1:600, ab3328, Abcam, Cambridge, UK). After another washing step, slides were incubated with rabbit-polymer coupled to alkaline phosphatase (Biocare) for 30 min and washed again. Vulcan Fast Red (Biocare) was used as substrate and incubated for 12 min. Hematoxylin counterstaining was performed, and slides were dehydrated and mounted in Eukitt® (Sigma-Aldrich). Slides were evaluated using a MIRAX scanning system (Zeiss, Oberkochen, Germany).

20S proteasome isolation from mouse lungs: Isolation and purification of proteasomes from lung tissue was performed essentially as described by Dahlmann et al.[4], except for the fact that DEAE-Toyopearl was used for the initial step of anion exchange chromatography and Superose 6 instead of Sepharose for gel chromatography. After chromatography on arginine-Sepharose, the enzyme preparation was concentrated by ultracentrifugation and the

precipitate dissolved in TSDG buffer (10 mM Tris/HCl, 25 mM KCl, 1.1 mM $MgCl_2$, 0.1 mM EDTA, 1 mM DTT, 1 mM NaN_3, 10% glycerol, pH 7) containing 2 mM ATP. 20S and 26S proteasomes were then separated by centrifugation in a glycerol gradient (20% - 40% dissolved in TSDG buffer). Centrifugation was performed for 24 h at 25,000 rpm in a Beckman SW28 rotor and afterwards the gradient was fractionated into fractions of 0.5 ml.

Determination of proteolytic activity was performed by use of fluorogenic peptide substrates as described by Dahlmann et al.[5].

For detection of proteasome activity by substrate overlay technique after non-denaturing polyacrylamide gel electrophoresis, the substrate Bz-VGR-MCA was used. This technique as well as non-equilibrium pH gradient and SDS-PAGE were performed as described by Dahlmann et al.[4].

2D gel electrophoresis / In-gel tryptic digest: Purified 20S proteasomes were sepaprated by two dimensional gel electrophoresis (protalys, Berlin, Germany) (1D: Nonequilibrium pH gel electrophoresis-IEF; 2D 2.6-15% SDS-polyacrylamide gel electrophoresis). After visualization of proteins (Colloidal Coomassie-G 250 staining), gel spots were excised for in-gel tryptic digest. Destaining of gel spots was done by washing in 200 µl nanopure water followed by dehydration in 200 µl 60% acetonitrile (each 3 x 10 minutes or until gel spots were completely destained). Acetonitrile was then removed and gel spots were rehydrated in 10 µl digestion-buffer (1 mM Tris-HCl, pH 7.5) containing 0.01 µg/µl trypsin (Sequencing grade Modified Trypsin; Promega). Protein samples were digested over-night at 37°C.

Mass Spectrometry: Proteins were identified using Matrix-assisted laser desorption ionization time-of-flight (MALDI-TOF) or liquid chromatrography electrospray-based (LC-MS/MS) mass spectrometry. Peptide mass fingerprints were obtained on a MALDI-TOF/TOF tandem mass spectrometer (ABI 4700 ProteomicsAnalyzer, Applied Biosystems)[6]. The tryptic digest was diluted with one equivalent of MALDI matrix consisting of

2,5-dihydroxy-benzoic acid (Sigma-Aldrich) (20 mg/ml in 20% acetonitrile, 0.1% TFA) and 2-hydroxy-5-bethoxybenzoic acid (Fluka) (20 mg/ml in 20% acetonitrile, 0.1% TFA) in a 9:1 ratio (v/v), and spotted onto a steel target plate. Peptide mass fingerprint identification of the sample protein was done by comparing peptide masses of the tryptic digest to the virtually trypsinized Ensembl Mouse protein database (database downloaded from www.ensembl.org). The database search was performed using the MASCOT Database search engine v1.9 (Matrix Science Ltd.). Search parameter settings were 150 ppm peptide mass tolerance and one allowed missed cleavage. LC-MS/MS analysis was performed on an Ultimate3000 nano HPLC system (Dionex, Sunnyvale, CA) coupled to a LTQ OrbitrapXL mass spectrometer (Thermo Fisher Scientific) by a nano spray ion source. Samples from in-gel digest were acidified using TFA and automatically loaded to the HPLC system as described by Hauck et al.[7]. The acquired spectra (Thermo raw file) were exported to Mascot Deamon and searched against the Ensembl_Mouse protein database. Search parameters included fixed modification Carbamidomethyl (C) and variable modifications Deaminated (NQ) and Oxidation (M). Peptide tolerance was set to 10 ppm and MS/MS tolerance to 0.6 Da. Only 2, 3 and 4 fold charged peptides were selected for protein identification. Search results were viewed using the Scaffold software (Scaffold 3).

SUPPLEMENT METHODS REFERENCES

1. Burgstaller, G., Oehrle, B., Koch, I., Lindner, M. & Eickelberg, O. Multiplex profiling of cellular invasion in 3D cell culture models. *PLoS ONE* **8,** e63121 (2013).
2. Königshoff, M. *et al.* WNT1-inducible signaling protein-1 mediates pulmonary fibrosis in mice and is upregulated in humans with idiopathic pulmonary fibrosis. *J. Clin. Invest.* **119,** 772–787 (2009).

3. Van Rijt, S. H. *et al.* Acute cigarette smoke exposure impairs proteasome function in the lung. *Am. J. Physiol. Lung Cell Mol. Physiol.* **303,** L814–823 (2012).
4. Dahlmann, B., Kuehn, L. & Reinauer, H. Studies on the activation by ATP of the 26 S proteasome complex from rat skeletal muscle. *Biochem. J.* **309 (Pt 1),** 195–202 (1995).
5. Dahlmann, B., Ruppert, T., Kuehn, L., Merforth, S. & Kloetzel, P. M. Different proteasome subtypes in a single tissue exhibit different enzymatic properties. *J. Mol. Biol* **303,** 643–653 (2000).
6. Hauck, S. M. *et al.* Retinal Mueller glial cells trigger the hallmark inflammatory process in autoimmune uveitis. *J. Proteome Res.* **6,** 2121–2131 (2007).
7. Hauck, S. M. *et al.* Deciphering membrane-associated molecular processes in target tissue of autoimmune uveitis by label-free quantitative mass spectrometry. *Mol. Cell Proteomics* **9,** 2292–2305 (2010).

SUPPLEMENTARY FIGURES

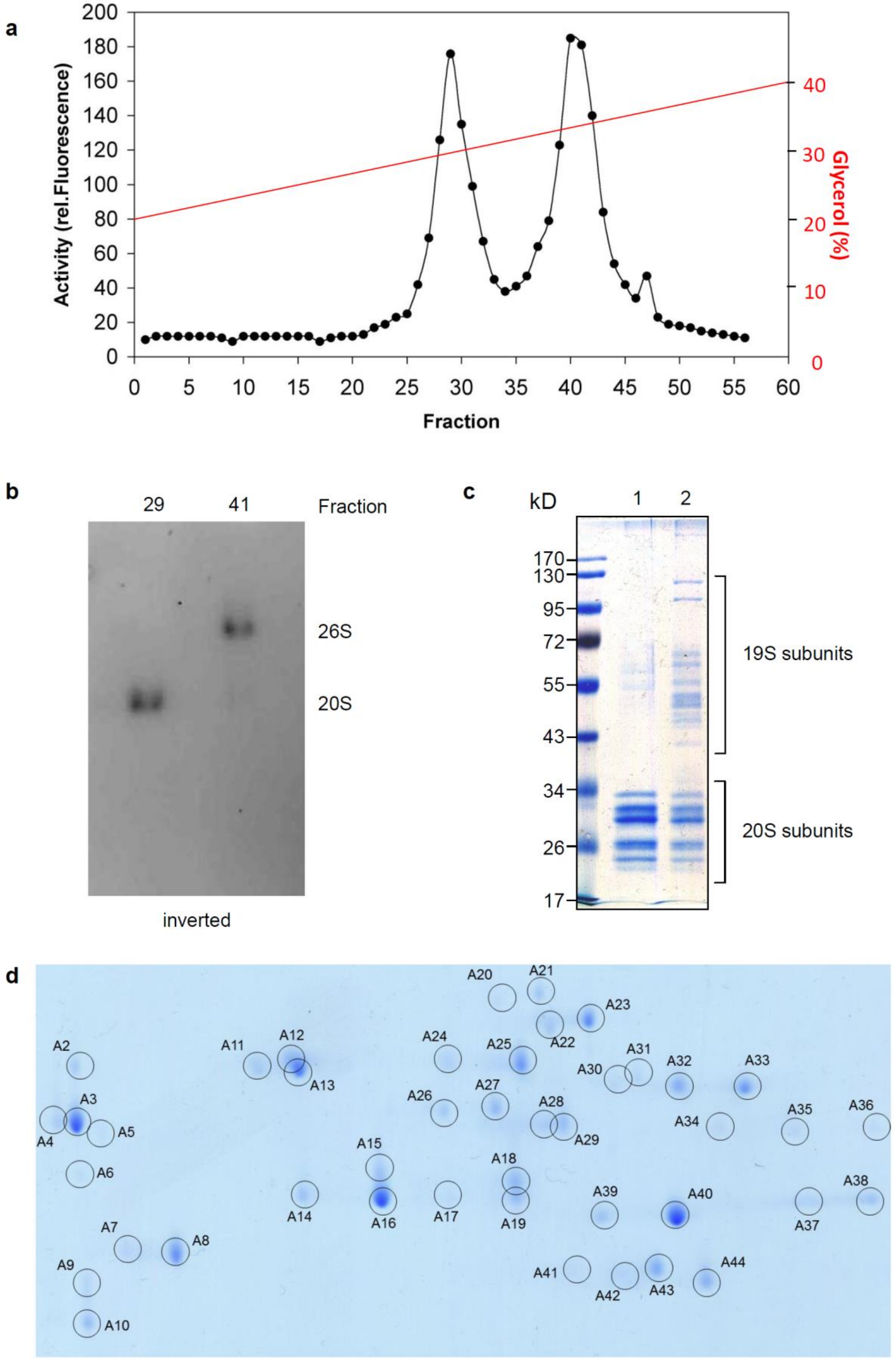

Figure S1. Glycerol gradient analysis to isolate 20S proteasomes from mouse lung for mass spectrometry. (a) Proteasomes were subjected to glycerol gradient centrifugation and afterwards the gradient was fractionated into fractions of 0.5 ml. In each fraction, proteasome activity was measured by use of Suc-LLVY-MCA as substrate to detect the chymotrypsin-like activity of the proteasome. (b) Native PAGE of glycerol gradient fractions 29 and 41: 20 µl of each were subjected to non-denaturing PAGE and after the run proteasome activity was detected by substrate overlay technique (Suc-LLVY-MCA). (c) Coomassie stained SDS-PAGE of 8 µg of each 20S proteasome (lane 1) or 26S proteasome (lane 2) separated by glycerol gradient centrifugation. (d) Coomassie stained 2D gel with annotated spots for mass-spectrometry protein identification (for protein identities, see Supplementary Table S2).

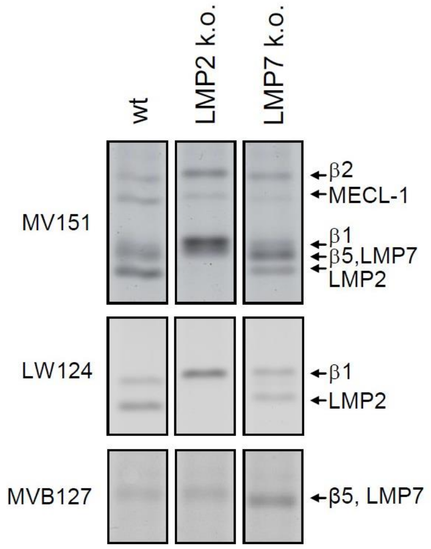

Figure S2. Labeling specificities of activity-based probes. Native splenocyte lysates of wildtype, LMP2 k.o. or LMP7 k.o. mice were labeled with activity-based probes MV151 (labeling all active β-subunits), LW124 (β1 and LMP2 specific) or MVB127 (β5 and LMP7 specific) and separated by SDS-PAGE. Bands originate from the same gel. The molecular weight of mouse β5 and mouse LMP7 is very similar and thus, these subunits separate only marginally on an SDS gel.

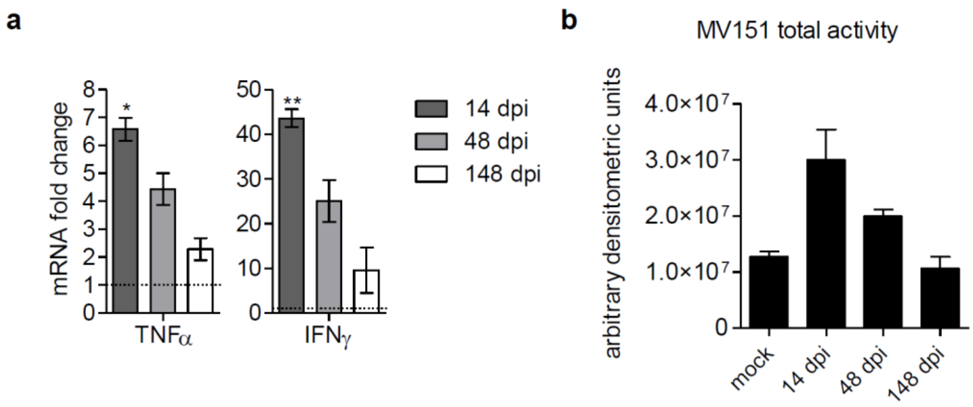

Figure S3. Characterization of MHV68-infected mouse lungs. (a) mRNA levels of inflammatory cytokines TNFα and IFNγ during course of MHV-68 infection displayed as fold over uninfected controls, Rpl19 served as housekeeping gene (mean +/- SEM, Kruskal-Wallis Test with Dunn's Post Test, * = $p<0.05$, ** = $p<0.01$). (b) Densitometric analysis of total proteasome activity of ABP MV151 labeled native lung lysates of uninfected mice or MHV-68 infected mice (day 14, 48 and 148). (mean +/- SEM, Kruskal-Wallis Test with Dunn's Post Test, * = $p<0.05$, ** = $p<0.01$).

SUPPLEMENTARY TABLES

Table S1: Primer sequences

**Name**	**Acc. No.**	**Forward Primer (5‘-3‘)**	**Reverse Primer (5‘-3‘)**
**Mouse**			
**Nlrc5**	NM_001033207.3	AGGCTCCCACTGCTTAGACA	CGGACAGCAAGAGTTTCTCC
**Rpl19**	NM_001159483.1	CGGGAATCCAAGAAGATTGA	TTCAGCTTGTGGATGTGCTC
**Psma3**	NM_011184.4	TGAAGAAGGCTCCAATAAACGTCT	AACGAGCATCTGCCAGCAA
**Psmb5**	NM_011186.1	TGCTCGCTAACATGGTGTATCAGTA	GGCCTCTCTTATCCCAGCCA
**Psmb6**	NM_008946.4	AGACGCTGTCACTTACCAACTTGG	AAGAGACTGGCGGCTGTGTG
**Psmb7**	NM_011187.1	TGCCTTATGTCACCATGGGTTC	TTCCTCCTCCATATCTGGCCTAA
**Psmb8**	NM_010724.2	TGCTTATGCTACCCACAGAGACAA	TTCACTTTCACCCAACCGTC
**Psmb9**	NM_013585.2	GTACCGTGAGGACTTGTTAGCGC	GGCTGTCGAATTAGCATCCCT
**Psmb10**	NM_013640.3	GAAGACCGGTTCCAGCCAA	CACTCAGGATCCCTGCTGTGAT
**Tnf**	NM_013693.3	CATCTTCTCAAAATTCGAGTGACAA	TGGGAGTAGACAAGGTACAACCC
**Ifng**	NM_008337.3	ACGGCACAGTCATTGAAAGCCTA	GTCACCATCCTTTTGCCAGTTCC
**Human**			
**NLRC5**	NM_032206.4	CTGCAGCCAAGTTCTTAGGG	TCAGCTGAGGGAGTTGAGGT
**RPL19**	NM_000981.3	GAGACCAATGAAATCGCCAATG	GCGGATGATCAGCCCATCTT
**PSMA3**	NM_002788.3	ACAGTGTGAATGACGGTGCG	GCAGCTTGCCTGGCTTTG
**PSMB5**	NM_002797.4	AGGAATCGAAATGCTTCATGGA	GTAAGCACCCGCTGTAGCCC
**PSMB6**	NM_002798.2	ACACCTATTCACGACCGCATTT	GTAGGTGACAGCATCAGCTACTGC
**PSMB7**	NM_002799.3	CTTCAACGACCTGGGCTCC	TCTTGTTGGGCACTGTGTATGG
**PSMB8**	NM_148919.3	AGTACTGGGAGCGCCTGCT	CCGACACTGAAATACGTTCTCCA
**PSMB9**	NM_002800.4	CGTTGTGATGGGTTCTGATTCC	GACAGCTTGTCAAACACTCGGTT
**PSMB10**	NM_002801.3	TGCTGCGGACACTGAGCTC	GCTGTGGTTCCAGGCACAAA

Table S2: Mass spectrometry analysis of 2D gel excised spots

spot ID	Protein Name	Accession Number	Protein MW	Peptide Count	Protein Score	Total Ion Score	Best Ion Score	Total MS Ion Cluster Area	MS Ion Cluster Area Matched %
**A2**	Psma5|MGI (curated)|Proteasome subunit alpha type-5 (EC 3.4.25.1)(Proteasome zeta chain)(Macropain zeta chain)(Multicatalytic endopeptidase complex zeta chain) [Source:UniProtKB/Swiss-Prot;Acc:Q9Z2U1]|3|ENSMUSG00000068749|ENSMUST00000090569	ENSMUSP00000088057	26565	6	95	51	51	363064	26
**A3**	Psma5|MGI (curated)|Proteasome subunit alpha type-5 (EC 3.4.25.1)(Proteasome zeta chain)(Macropain zeta chain)(Multicatalytic endopeptidase complex zeta chain) [Source:UniProtKB/Swiss-Prot;Acc:Q9Z2U1]|3|ENSMUSG00000068749|ENSMUST00000090569	ENSMUSP00000088057	26565	7	345	286	123	1119377	37
**A4**	Psma5|MGI (curated)|Proteasome subunit alpha type-5 (EC 3.4.25.1)(Proteasome zeta chain)(Macropain zeta chain)(Multicatalytic endopeptidase complex zeta chain) [Source:UniProtKB/Swiss-Prot;Acc:Q9Z2U1]|3|ENSMUSG00000068749|ENSMUST00000090569	ENSMUSP00000088057	26565	7	205	150	79	328137	34
**A5**	Psma5|MGI (curated)|Proteasome subunit alpha type-5 (EC 3.4.25.1)(Proteasome zeta chain)(Macropain zeta chain)(Multicatalytic endopeptidase complex zeta chain) [Source:UniProtKB/Swiss-Prot;Acc:Q9Z2U1]|3|ENSMUSG00000068749|ENSMUST00000090569	ENSMUSP00000088057	26565	7	246	186	76	149782	42
**A6**	Psma5|MGI (curated)|Proteasome subunit alpha type-5 (EC 3.4.25.1)(Proteasome zeta chain)(Macropain zeta chain)(Multicatalytic endopeptidase complex zeta chain) [Source:UniProtKB/Swiss-Prot;Acc:Q9Z2U1]|3|ENSMUSG00000068749|ENSMUST00000090569	ENSMUSP00000088057	26565	5	228	196	86	135406	32
**A7**	Psmb6|MGI (curated)|Proteasome subunit beta type-6 Precursor (EC 3.4.25.1)(Proteasome delta chain)(Macropain delta chain)(Multicatalytic endopeptidase complex delta chain)(Proteasome subunit Y) [Source:UniProtKB/Swiss-Prot;Acc:Q60692]|11|ENSMUSG0000	ENSMUSP00000018430	25591	8	126	55	55	439117	43
**A9**	Psmb9|MGI (automatic)|Proteasome subunit beta type-9 Precursor (EC 3.4.25.1)(Proteasome subunit beta-1i)(Proteasome chain 7)(Macropain chain 7)(Multicatalytic endopeptidase complex chain 7)(RING12 protein)(Low molecular mass protein 2)(LMP-2d) [Sour	ENSMUSP00000075907	23482	5	130	95	77	225749	27
**A11**	Psma3|MGI (automatic)|Proteasome subunit alpha type-3 (EC 3.4.25.1)(Proteasome component C8)(Macropain subunit C8)(Multicatalytic endopeptidase complex subunit C8)(Proteasome subunit K) [Source:UniProtKB/Swiss-Prot;Acc:O70435]|12|ENSMUSG00000060073|	ENSMUSP00000071624	28615	12	321	226	61	459544	28
**A12**	Psma3|MGI (automatic)|Proteasome subunit alpha type-3 (EC 3.4.25.1)(Proteasome component C8)(Macropain subunit C8)(Multicatalytic endopeptidase complex subunit C8)(Proteasome subunit K) [Source:UniProtKB/Swiss-Prot;Acc:O70435]|12|ENSMUSG00000060073|	ENSMUSP00000071624	28615	11	411	327	96	924322	25
**A13**	Psma3|MGI (automatic)|Proteasome subunit alpha type-3 (EC 3.4.25.1)(Proteasome component C8)(Macropain subunit C8)(Multicatalytic endopeptidase complex subunit C8)(Proteasome subunit K) [Source:UniProtKB/Swiss-Prot;Acc:O70435]|12|ENSMUSG00000060073|	ENSMUSP00000071624	28615	11	399	316	103	1785503	28
**A14**	Psmb4|MGI (curated)|Proteasome subunit beta type-4 Precursor (Proteasome beta chain)(EC 3.4.25.1)(Macropain beta chain)(Multicatalytic endopeptidase complex beta chain)(Proteasome chain 3) [Source:UniProtKB/Swiss-Prot;Acc:P99026]|3|ENSMUSG0000000577	ENSMUSP00000005923	29211	7	267	222	73	726792	34
**A15**	Psmb4|MGI (curated)|Proteasome subunit beta type-4 Precursor (Proteasome beta chain)(EC 3.4.25.1)(Macropain beta chain)(Multicatalytic endopeptidase complex beta chain)(Proteasome chain 3) [Source:UniProtKB/Swiss-Prot;Acc:P99026]|3|ENSMUSG0000000577	ENSMUSP00000005923	29211	8	241	184	92	521272	32
**A16**	Psmb4|MGI (curated)|Proteasome subunit beta type-4 Precursor (Proteasome beta chain)(EC 3.4.25.1)(Macropain beta chain)(Multicatalytic endopeptidase complex beta chain)(Proteasome chain 3) [Source:UniProtKB/Swiss-Prot;Acc:P99026]|3|ENSMUSG0000000577	ENSMUSP00000005923	29211	8	286	228	146	1916248	59
**A17**	Psmb3|MGI (curated)|Proteasome subunit beta type-3 (EC 3.4.25.1)(Proteasome theta chain)(Proteasome chain 13)(Proteasome component C10-II) [Source:UniProtKB/Swiss-Prot;Acc:Q9R1P1]|11|ENSMUSG00000069744|ENSMUST00000103147	ENSMUSP00000099436	23235	7	120	77	52	270844	13
**and**	Psmb4|MGI (curated)|Proteasome subunit beta type-4 Precursor (Proteasome beta chain)(EC 3.4.25.1)(Macropain beta chain)(Multicatalytic endopeptidase complex beta chain)(Proteasome chain 3) [Source:UniProtKB/Swiss-Prot;Acc:P99026]|3|ENSMUSG0000000577	ENSMUSP00000005923	29211	6	102	65	27	270844	20
**A20**	Psma1|MGI (curated)|Proteasome subunit alpha type-1 (EC 3.4.25.1)(Proteasome component C2)(Macropain subunit C2)(Multicatalytic endopeptidase complex subunit C2)(Proteasome nu chain) [Source:UniProtKB/Swiss-Prot;Acc:Q9R1P4]|7|ENSMUSG00000030751|ENSM	ENSMUSP00000033008	29813	6	116	76	41	131208	54
**A21**	Psma1|MGI (curated)|Proteasome subunit alpha type-1 (EC 3.4.25.1)(Proteasome component C2)(Macropain subunit C2)(Multicatalytic endopeptidase complex subunit C2)(Proteasome nu chain) [Source:UniProtKB/Swiss-Prot;Acc:Q9R1P4]|7|ENSMUSG00000030751|ENSM	ENSMUSP00000033008	29813	9	223	161	92	492981	50
**A22**	Psma1|MGI (curated)|Proteasome subunit alpha type-1 (EC 3.4.25.1)(Proteasome component C2)(Macropain subunit C2)(Multicatalytic endopeptidase complex subunit C2)(Proteasome nu chain) [Source:UniProtKB/Swiss-Prot;Acc:Q9R1P4]|7|ENSMUSG00000030751|ENSM	ENSMUSP00000033008	29813	11	329	247	109	960792	74
**A23**	Psma1|MGI (curated)|Proteasome subunit alpha type-1 (EC 3.4.25.1)(Proteasome component C2)(Macropain subunit C2)(Multicatalytic endopeptidase complex subunit C2)(Proteasome nu chain) [Source:UniProtKB/Swiss-Prot;Acc:Q9R1P4]|7|ENSMUSG00000030751|ENSM	ENSMUSP00000033008	29813	11	343	260	126	1488460	69
**A24**	Psmb7|MGI (curated)|Proteasome subunit beta type-7 Precursor (EC 3.4.25.1)(Proteasome subunit Z)(Macropain chain Z)(Multicatalytic endopeptidase complex chain Z) [Source:UniProtKB/Swiss-Prot;Acc:P70195]|2|ENSMUSG00000026750|ENSMUST00000028083	ENSMUSP00000028083	30214	7	204	150	58	563024	50

spot ID	Protein Name	Accession Number	Protein MW	Peptide Count	Protein Score	Total Ion Score	Best Ion Score	Total MS Ion Cluster Area	MS Ion Cluster Area Matched %
**and**	Psmb10|MGI (automatic)|Proteasome subunit beta type-10 Precursor (EC 3.4.25.1)(Proteasome subunit beta-2i)(Proteasome MECl-1)(Macropain subunit MECl-1)(Multicatalytic endopeptidase complex subunit MECl-1) [Source:UniProtKB/Swiss-Prot;Acc:O35955]|8|E	ENSMUSP00000034369	29330	1	48	44	44	563024	4
**A25**	Psmb7|MGI (curated)|Proteasome subunit beta type-7 Precursor (EC 3.4.25.1)(Proteasome subunit Z)(Macropain chain Z)(Multicatalytic endopeptidase complex chain Z) [Source:UniProtKB/Swiss-Prot;Acc:P70195]|2|ENSMUSG00000026750|ENSMUST00000028083	ENSMUSP00000028083	30214	10	293	209	69	1700471	57
**and**	Psmb10|MGI (automatic)|Proteasome subunit beta type-10 Precursor (EC 3.4.25.1)(Proteasome subunit beta-2i)(Proteasome MECl-1)(Macropain subunit MECl-1)(Multicatalytic endopeptidase complex subunit MECl-1) [Source:UniProtKB/Swiss-Prot;Acc:O35955]|8|E	ENSMUSP00000034369	29330	1	48	45	45	1700471	4
**and**	Psmb10|MGI (automatic)|Proteasome subunit beta type-10 Precursor (EC 3.4.25.1)(Proteasome subunit beta-2i)(Proteasome MECl-1)(Macropain subunit MECl-1)(Multicatalytic endopeptidase complex subunit MECl-1) [Source:UniProtKB/Swiss-Prot;Acc:O35955]|8|E	ENSMUSP00000034369	29330	9	415	340	185	1365419	59
**A28**	Psma6|MGI (automatic)|Proteasome subunit alpha type-6 (EC 3.4.25.1)(Proteasome iota chain)(Macropain iota chain)(Multicatalytic endopeptidase complex iota chain) [Source:UniProtKB/Swiss-Prot;Acc:Q9QUM9]|12|ENSMUSG00000021024|ENSMUST00000021412	ENSMUSP00000021412	27811	4	66	47	47	1365419	6
**A29**	Psma6|MGI (automatic)|Proteasome subunit alpha type-6 (EC 3.4.25.1)(Proteasome iota chain)(Macropain iota chain)(Multicatalytic endopeptidase complex iota chain) [Source:UniProtKB/Swiss-Prot;Acc:Q9QUM9]|12|ENSMUSG00000021024|ENSMUST00000021412	ENSMUSP00000021412	27811	8	250	198	82	496373	52
**and**	Psma6|MGI (automatic)|Proteasome subunit alpha type-6 (EC 3.4.25.1)(Proteasome iota chain)(Macropain iota chain)(Multicatalytic endopeptidase complex iota chain) [Source:UniProtKB/Swiss-Prot;Acc:Q9QUM9]|12|ENSMUSG00000021024|ENSMUST00000021412	ENSMUSP00000021412	27811	10	327	256	80	654306	61
**A30**	Mcm2|MGI (automatic)|DNA replication licensing factor MCM2 (Minichromosome maintenance protein 2 homolog)(Nuclear protein BM28) [Source:UniProtKB/Swiss-Prot;Acc:P97310]|6|ENSMUSG00000002870|ENSMUST00000058011	ENSMUSP00000061923	102698	10	66	44	44	654306	37
**and**	Psma4|MGI (automatic)|Proteasome subunit alpha type-4 (EC 3.4.25.1)(Proteasome component C9)(Macropain subunit C9)(Multicatalytic endopeptidase complex subunit C9)(Proteasome subunit L) [Source:UniProtKB/Swiss-Prot;Acc:Q9R1P0]|9|ENSMUSG00000032301|E	ENSMUSP00000034848	29737	6	180	143	66	123819	35
**A31**	Psma1|MGI (curated)|Proteasome subunit alpha type-1 (EC 3.4.25.1)(Proteasome component C2)(Macropain subunit C2)(Multicatalytic endopeptidase complex subunit C2)(Proteasome nu chain) [Source:UniProtKB/Swiss-Prot;Acc:Q9R1P4]|7|ENSMUSG00000030751|ENSM	ENSMUSP00000033008	29813	6	62	25	25	123819	21
**A32**	Psma1|MGI (curated)|Proteasome subunit alpha type-1 (EC 3.4.25.1)(Proteasome component C2)(Macropain subunit C2)(Multicatalytic endopeptidase complex subunit C2)(Proteasome nu chain) [Source:UniProtKB/Swiss-Prot;Acc:Q9R1P4]|7|ENSMUSG00000030751|ENSM	ENSMUSP00000033008	29813	10	229	159	71	244083	52
**and**	Psma4|MGI (automatic)|Proteasome subunit alpha type-4 (EC 3.4.25.1)(Proteasome component C9)(Macropain subunit C9)(Multicatalytic endopeptidase complex subunit C9)(Proteasome subunit L) [Source:UniProtKB/Swiss-Prot;Acc:Q9R1P0]|9|ENSMUSG00000032301|E	ENSMUSP00000034848	29737	7	258	217	82	491609	39
**A33**	Psma1|MGI (curated)|Proteasome subunit alpha type-1 (EC 3.4.25.1)(Proteasome component C2)(Macropain subunit C2)(Multicatalytic endopeptidase complex subunit C2)(Proteasome nu chain) [Source:UniProtKB/Swiss-Prot;Acc:Q9R1P4]|7|ENSMUSG00000030751|ENSM	ENSMUSP00000033008	29813	3	47	34	34	491609	8
**A34**	Psma4|MGI (automatic)|Proteasome subunit alpha type-4 (EC 3.4.25.1)(Proteasome component C9)(Macropain subunit C9)(Multicatalytic endopeptidase complex subunit C9)(Proteasome subunit L) [Source:UniProtKB/Swiss-Prot;Acc:Q9R1P0]|9|ENSMUSG00000032301|E	ENSMUSP00000034848	29737	8	381	331	85	730769	41
**and**	Psma7|MGI (curated)|Proteasome subunit alpha type-7 (EC 3.4.25.1)(Proteasome subunit RC6-1) [Source:UniProtKB/Swiss-Prot;Acc:Q9Z2U0]|2|ENSMUSG00000027566|ENSMUST00000029082	ENSMUSP00000029082	28009	9	204	127	61	88216	44
**A35**	Psma1|MGI (curated)|Proteasome subunit alpha type-1 (EC 3.4.25.1)(Proteasome component C2)(Macropain subunit C2)(Multicatalytic endopeptidase complex subunit C2)(Proteasome nu chain) [Source:UniProtKB/Swiss-Prot;Acc:Q9R1P4]|7|ENSMUSG00000030751|ENSM	ENSMUSP00000033008	29813	3	70	54	34	88216	22
**A37**	Psma7|MGI (curated)|Proteasome subunit alpha type-7 (EC 3.4.25.1)(Proteasome subunit RC6-1) [Source:UniProtKB/Swiss-Prot;Acc:Q9Z2U0]|2|ENSMUSG00000027566|ENSMUST00000029082	ENSMUSP00000029082	28009	9	188	116	77	95074	40
**A38**	Psmb1|MGI (automatic)|Proteasome subunit beta type-1 Precursor (EC 3.4.25.1)(Proteasome component C5)(Macropain subunit C5)(Multicatalytic endopeptidase complex subunit C5)(Proteasome gamma chain) [Source:UniProtKB/Swiss-Prot;Acc:O09061]|17|ENSMUSG0	ENSMUSP00000014913	26583	11	381	288	106	491567	68
**A39**	Psmb1|MGI (automatic)|Proteasome subunit beta type-1 Precursor (EC 3.4.25.1)(Proteasome component C5)(Macropain subunit C5)(Multicatalytic endopeptidase complex subunit C5)(Proteasome gamma chain) [Source:UniProtKB/Swiss-Prot;Acc:O09061]|17|ENSMUSG0	ENSMUSP00000014913	26583	11	377	284	103	698770	69
**A40**	Psma2|MGI (automatic)|Proteasome subunit alpha type-2 (EC 3.4.25.1)(Proteasome component C3)(Macropain subunit C3)(Multicatalytic endopeptidase complex subunit C3) [Source:UniProtKB/Swiss-Prot;Acc:P49722]|13|ENSMUSG00000015671|ENSMUST00000082305	ENSMUSP00000106140	26024	7	246	186	97	458855	45
**A41**	Psma2|MGI (automatic)|Proteasome subunit alpha type-2 (EC 3.4.25.1)(Proteasome component C3)(Macropain subunit C3)(Multicatalytic endopeptidase complex subunit C3) [Source:UniProtKB/Swiss-Prot;Acc:P49722]|13|ENSMUSG00000015671|ENSMUST00000082305	ENSMUSP00000106140	26024	9	430	350	146	1490688	66

spot ID	Protein Name	Accession Number	Protein MW	Peptide Count	Protein Score	Total Ion Score	Best Ion Score	Total MS Ion Cluster Area	MS Ion Cluster Area Matched %
and	Psmb2|MGI (curated)|Proteasome subunit beta type-2 (EC 3.4.25.1)(Proteasome component C7-I)(Macropain subunit C7-I)(Multicatalytic endopeptidase complex subunit C7-I) [Source:UniProtKB/Swiss-Prot;Acc:Q9R1P3]|4|ENSMUSG00000028837|ENSMUST00000030642	ENSMUSP00000030642	23063	6	218	176	79	359746	49
A42	Psmb8|MGI (automatic)|Proteasome subunit beta type-8 Precursor (EC 3.4.25.1)(Proteasome subunit beta-5i)(Proteasome component C13)(Macropain subunit C13)(Multicatalytic endopeptidase complex subunit C13) [Source:UniProtKB/Swiss-Prot;Acc:P28063]|17|E	ENSMUSP00000025196	30526	8	139	87	62	359746	14
and	Psmb8|MGI (automatic)|Proteasome subunit beta type-8 Precursor (EC 3.4.25.1)(Proteasome subunit beta-5i)(Proteasome component C13)(Macropain subunit C13)(Multicatalytic endopeptidase complex subunit C13) [Source:UniProtKB/Swiss-Prot;Acc:P28063]|17|E	ENSMUSP00000025196	30526	10	218	131	86	387945	47
A43	Psmb2|MGI (curated)|Proteasome subunit beta type-2 (EC 3.4.25.1)(Proteasome component C7-I)(Macropain subunit C7-I)(Multicatalytic endopeptidase complex subunit C7-I) [Source:UniProtKB/Swiss-Prot;Acc:Q9R1P3]|4|ENSMUSG00000028837|ENSMUST00000030642	ENSMUSP00000030642	23063	5	149	118	73	387945	19
A44	Psmb2|MGI (curated)|Proteasome subunit beta type-2 (EC 3.4.25.1)(Proteasome component C7-I)(Macropain subunit C7-I)(Multicatalytic endopeptidase complex subunit C7-I) [Source:UniProtKB/Swiss-Prot;Acc:Q9R1P3]|4|ENSMUSG00000028837|ENSMUST00000030642	ENSMUSP00000030642	23063	11	612	503	174	1853259	70
and	Psmb8|MGI (automatic)|Proteasome subunit beta type-8 Precursor (EC 3.4.25.1)(Proteasome subunit beta-5i)(Proteasome component C13)(Macropain subunit C13)(Multicatalytic endopeptidase complex subunit C13) [Source:UniProtKB/Swiss-Prot;Acc:P28063]|17|E	ENSMUSP00000025196	30526	13	407	293	96	1260507	60
	Psmb5|MGI (automatic)|Proteasome subunit beta type-5 Precursor (EC 3.4.25.1)(Proteasome epsilon chain)(Macropain epsilon chain)(Multicatalytic endopeptidase complex epsilon chain)(Proteasome subunit X)(Proteasome chain 6) [Source:UniProtKB/Swiss-Pro	ENSMUSP00000107118	22645	3	48	31	31	1260507	12

Spots not significantly identified by MALDI-MS were analyzed by LC-MS/MS:

spot ID	Protein Name	Accession Number	Protein MW	Peptide Count	Protein Score	Best MS/MS Ion Score	Sequence Coverage (%)
A8	Psmb6|MGI Symbol|proteasome (prosome, macropain) subunit, beta type 6 Gene [Source:MGI Symbol;Acc:MGI:104880]|ENSMUSG00000018286|11|ENSMUST00000018430	ENSMUSP00000018430	25591	12	3063	105	38
A10	Psmb9|MGI Symbol|proteasome (prosome, macropain) subunit, beta type 9 (large multifunctional peptidase 2) Gene [Source:MGI Symbol;Acc:MGI:1346526]|ENSMUSG00000024337|17|ENSMUST00000076602	ENSMUSP00000075907	23482	15	2592	118	40
A18	Psmb3|MGI Symbol|proteasome (prosome, macropain) subunit, beta type 3 Gene [Source:MGI Symbol;Acc:MGI:1347014]|ENSMUSG00000069744|11|ENSMUST00000103147	ENSMUSP00000099436 und	23235	27	4586	121	60
A18	Psmb9|MGI Symbol|proteasome (prosome, macropain) subunit, beta type 9 (large multifunctional peptidase 2) Gene [Source:MGI Symbol;Acc:MGI:1346526]|ENSMUSG00000024337|17|ENSMUST00000076602	ENSMUSP00000075907	23482	8	555	107	33
A19	Psmb3|MGI Symbol|proteasome (prosome, macropain) subunit, beta type 3 Gene [Source:MGI Symbol;Acc:MGI:1347014]|ENSMUSG00000069744|11|ENSMUST00000103147	ENSMUSP00000099436 und	23235	39	4577	122	61
A19	Psma6|MGI Symbol|proteasome (prosome, macropain) subunit, alpha type 6 Gene [Source:MGI Symbol;Acc:MGI:1347006]|ENSMUSG00000021024|12|ENSMUST00000021412	ENSMUSP00000021412	27811	5	218	94	21
A26	Psma6|MGI Symbol|proteasome (prosome, macropain) subunit, alpha type 6 Gene [Source:MGI Symbol;Acc:MGI:1347006]|ENSMUSG00000021024|12|ENSMUST00000021412	ENSMUSP00000021412 und	27811	12	899	116	41
A26	Psma3|MGI Symbol|proteasome (prosome, macropain) subunit, alpha type 3 Gene [Source:MGI Symbol;Acc:MGI:104883]|ENSMUSG00000060073|12|ENSMUST00000160027	ENSMUSP00000125548	28615	14	740	114	28
A35	Psma7|MGI Symbol|proteasome (prosome, macropain) subunit, alpha type 7 Gene [Source:MGI Symbol;Acc:MGI:1347070]|ENSMUSG00000027566|2|ENSMUST00000029082	ENSMUSP00000029082 und	28009	21	1892	130	48
A35	Psma1|MGI Symbol|proteasome (prosome, macropain) subunit, alpha type 1 Gene [Source:MGI Symbol;Acc:MGI:1347005]|ENSMUSG00000030751|7|ENSMUST00000033008	ENSMUSP00000033008	29813	19	850	90	34
